# Delirium Screening in the Emergency Department: Barriers, Enablers and Alignment With Clinical Standards—A Mixed‐Methods Study

**DOI:** 10.1111/1742-6723.70303

**Published:** 2026-06-17

**Authors:** Barbara Zangerl, Jared‐Tyrell Ah‐Leong, Joshua Wilcox, Michelle Jory, Faye Jordan

**Affiliations:** ^1^ The Prince Charles Hospital Chermside Queensland Australia; ^2^ Queensland University of Technology Kelvin Grove Queensland Australia; ^3^ Northside Clinical School The University of Queensland Chermside Queensland Australia

**Keywords:** aged, Australia, delirium, emergency service, hospital, mass screening

## Abstract

**Objective:**

Delirium is common and serious among older adults in emergency departments (EDs) yet screening often falls short of national expectations. This study evaluated current delirium screening practices in a metropolitan ED and identified barriers and enablers to implementing screening in line with the Australian Delirium Clinical Care Standard.

**Methods:**

A mixed‐methods quality assurance study was conducted in The Prince Charles Hospital ED. A retrospective audit of 238 medical records for patients aged ≥ 65 years examined the frequency of delirium screening and associated clinical or operational factors. Semi‐structured interviews with nine staff members explored perceptions, experiences and decision‐making processes related to screening. Deductive framework analysis guided integration of quantitative and qualitative data.

**Results:**

Of eligible patients, 4.2% were screened, with most assessments completed by specialist teams during weekday hours. Screened patients had longer ED stays, although this finding should be interpreted as exploratory. Nurses recognised major delirium risk factors but described screening as reactive rather than routine. Reported barriers included time pressures, environmental limitations, lack of digital integration and uncertainty about role responsibilities. Awareness of the Australian Delirium Clinical Care Standard was limited, contributing to inconsistent practice.

**Conclusions:**

Despite baseline knowledge of delirium risk, ED screening remains inconsistent and specialist‐dependent. Strengthening adherence to national standards requires embedding screening into routine nursing workflows through clearer role delineation and the integration of prompts to support systematic assessment for all older patients.

## 1 Introduction

Delirium is an acute neuropsychiatric syndrome affecting predominantly older adults and is associated with increased mortality, prolonged hospital stays and accelerated cognitive decline [1, 2, 3]. Despite its clinical importance, delirium remains markedly under‐recognised in emergency departments (EDs), where prevalence ranges from 8% to 40% among older patients and up to 85% of cases go undetected [4, 5, 6, 7]. Although digital screening tools have been introduced to improve detection [8], significant gaps in clinical recognition persist. Meged‐Book et al. reported that frontline staff identified delirium in fewer than 2% of cases, compared to 14.9% by trained evaluators [6]. This discrepancy underscored a persistent implementation gap between available screening approaches and routine ED practice.

In Australia, individuals aged 65 and older now account for at least 23% of all public emergency presentations; a cohort not only expected to continue to rise as the population ages but also at a higher risk of incident dementia, mortality and unplanned 28‐day readmission [9, 10]. Despite these risks, recognition of delirium has been historically poor, with formal documentation of delirium in medical records as low as 2% [11]. To improve these outcomes, the *Delirium Clinical Care Standard* mandates screening for patients aged ≥ 65 years (≥ 45 years for Aboriginal and Torres Strait Islander people) within 24 h of hospital presentation [12]. However, the extent to which this standard is implemented in routine ED care remains unclear, particularly in busy metropolitan environments where competing priorities and workflow challenges may limit uptake.

This mixed‐methods study addresses this gap by evaluating current delirium screening practices at a metropolitan ED, identifying barriers to implementation and assessing readiness for integrated digital screening tools into clinical workflow.

## 2 Methods

### 2.1 Design and Setting

We used a convergent mixed‐methods design at The Prince Charles Hospital (TPCH), Brisbane, a major tertiary referral centre. The study combined a retrospective chart audit with qualitative staff interviews to identify areas of practice convergence or conflict [13]. This approach was chosen to combine quantitative data describing current screening practices with qualitative interviews exploring the contextual factors and barriers influencing these practices. Reporting followed the Good Reporting of A Mixed Methods Study (GRAMMS) guidelines.

### 2.2 Theoretical Framework

A deductive approach [14], was applied to evaluate practice against Statements 1 and 4 of the Delirium Clinical Care Standard [12], focusing on early risk identification, validated screening and communication through interdisciplinary and digital solutions. This approach was selected to enable structured analysis of current practices against established national standards, providing a clear framework for identifying gaps between recommended and observed care. This framework informed the development of the interview guide and subsequent data analysis (Table S1).

### 2.3 Data Collection

The audit examined 10% of randomly selected records for patients aged ≥ 65 years from October 2024 (*n* = 250). This sampling approach was chosen to provide a feasible yet representative snapshot of routine screening practices. October was chosen as a typical month of ED activity as it is generally free from major service disruptions or the peak impact of seasonal illnesses, such as influenza, which can lead to atypical presentation patterns and complications from intercurrent illness. Delirium screening was identified through the presence of a documented clinical 4 A's Test (4AT; a brief validated screening tool for delirium assessing Alertness, Abbreviated mental state, Attention and Acute change or fluctuating course) in the patient's medical records or nursing progress notes. Variables included age, sex, arrival mode, Australasian Triage Scale (ATS) category, length of stay (LOS) in the ED, departure status and 4AT performance. Data were collected by three investigators (B.Z., J.A., M.J.) using a standardised data collection form. To ensure accuracy, a 10% subset was independently audited by a second investigator, with 100% agreement achieved across key variables.

In parallel, nine semi‐structured interviews were conducted with ED nurses in July 2025 using a thematic framework derived from Statements 1 and 4 of the *Delirium Clinical Care Standard*. Based on audit results, all nurses providing emergency care were invited to participate in the study on a first‐come‐first‐served basis. Interviews were guided by prompting questions informed by the thematic framework, audio‐recorded, transcribed verbatim and organised in a matrix format for systematic analysis. To ensure face validity, the interview guide was reviewed by two senior clinicians with expertise in emergency care and delirium prior to use. While formal piloting with the nursing cohort was not conducted, the guide's structure was strictly aligned with the established deductive framework of the national clinical standards to ensure relevance and clarity. To manage potential analytical bias, a clear separation of roles was maintained: interviews were conducted by one author (J.W.), while the primary data extraction and coding were performed independently by another (B.Z.), who was not present during the interview phase. This analysis followed a structured deductive approach in an iterative fashion, with coding patterns and thematic developments regularly discussed and independently assessed by the research team to reach a consensus. Furthermore, participant validation was employed; all participants were provided with their interview transcripts and a summary of the thematic analysis for verification and feedback, ensuring accuracy and credibility of the source data.

### 2.4 Data Analysis

In concordance with the principles of a convergent mixed‐methods approach, we analysed quantitative and qualitative data separately as outlined below. Results from both were subsequently compared and integrated in final themes.

#### 2.4.1 Statistical Analysis Quantitative Data

Records marked as ‘excluded’ (e.g., interhospital transfers, end‐of‐life care) were removed prior to analysis. ATS categories were grouped into two clinically relevant strata: ATS 1–2 (high acuity) and ATS 3–5 (low to moderate acuity). Descriptive statistics summarised patient characteristics. Quantitative data were analysed using Fisher's Exact Test for categorical variables (sex, mode of arrival, departure status, ATS group) and Welch's *t* test for continuous variables (age, ED LOS), with significance at *p* < 0.05.

#### 2.4.2 Thematic Analysis

Qualitative data underwent deductive thematic analysis following Braun and Clarke's principles, adapted for a deductive approach [15]. Data were organised in NVivo 14 to identify patterns and gaps between recommended and actual practice [16]. The codebook included node names, definitions, inclusion and exclusion criteria (Table S2), allowing query‐based exploration of patterns to verify coding consistency and examine relationships between themes [17]. To ensure a robust analysis, we distinguished between code saturation (the point at which the range of thematic issues was identified) and meaning saturation (the point at which the dimensions and nuances of these themes were fully understood) [18]. Comprehensive saturation was evidenced by each of the five qualitative codes being supported by 25–45 individual references across the nine interviews, with every participant providing at least two independent references per code (Table S3).

### 2.5 Ethical Considerations

This study was conducted as part of a quality assurance initiative under exemption from full ethical review by the Metro North Human Research Ethics Committee (EX/2024/MNH/113868). All participants provided informed consent prior to participation in the interviews.

## 3 Results

### 3.1 Chart Audit for Delirium Screening

Of the 250 patient records reviewed, 12 (5%) were excluded due to direct interhospital transfers (*n* = 8), admissions under emergency examination authority (*n* = 2) or presentation in a peri‐arrest state (*n* = 2). Among 238 patients included in the analysis, only 10 (4.2%) had documented delirium screening using the 4AT tool. Screening was conducted by the Fast Review and Integrated Liaison (FRAIL) Service—a specialist team focused on geriatric assessment—in eight cases (80%) and by ED nursing staff in two cases (20%). No screenings were recorded by medical officers.

Based on recorded ED arrival times, audited patient records were evenly distributed across time of the day (Table 1A) and day of the week (Table 1B). Screening occurred during weekday afternoon and evening hours, with no assessments recorded during night‐time or weekend hours. Outcomes of patients screened indicated that most (*n* = 7) did not show signs of cognitive impairment (4AT = 0), one patient had suggested cognitive impairment but was unlikely to have delirium (4AT = 1), and the remaining two patients had potential delirium (4AT = 4).

**TABLE 1:** Number of patient records audited and screened for delirium by time of the day (A) and day of the week (B).

A	Total records (*n*)	Screened (*n*)	Screening rate (%)
Morning (06:00–12:00)	64	2	3.1
Afternoon (12:00–17:00)	75	4	5.3
Evening (17:00–21:00)	53	4	7.6
Night (21:00–06:00)	46	0	0.0

B	Total records (*n*)	Screened (*n*)	Screening rate (%)
Monday	28	3	10.7
Tuesday	41	3	7.3
Wednesday	33	2	6.1
Thursday	45	2	4.4
Friday	32	0	0.0
Saturday	26	0	0.0
Sunday	33	0	0.0

Delirium screening was associated with longer ED stays (mean 380 vs. 305 min, Table 2, *p* = 0.013); however, given the small number of screened patients, this finding should be interpreted as exploratory. The same rationale applies to other observed differences, such as screened patients being older on average (82.3 years) compared with those not screened (78.5 years), and a higher proportion arrived by ambulance, while sex distribution was similar between groups (Table 2).

**TABLE 2:** Characteristics of patient records reviewed (*n* = 238) including estimate 95% confidence intervals (CI).

Variable	Category/measure	Screened (*n* = 10) Estimate (95% CI)	Not screened (*n* = 228) Estimate (95% CI)	*p*
Age in years	Mean	82.3 (76.6–88.0)	78.5 (77.4–79.7)	0.174
Sex (%)	Male (*n* = 97)	5.0 (2.0–10)	95.0 (90.0–98.0)	0.744
	Female (*n* = 141)	3.1 (0.6–8.8)	96.9 (91.2–99.4)	
Mode of arrival (%)	Ambulance (*n* = 160)	5.0 (2.2–9.6)	95 (90.4–97.8)	0.506
	Walk in (*n* = 78)	2.6 (0.3–9)	97.4 (91.99–99.7)	
Triage category (%)	1–2 (*n* = 68)	2.9 (0.4–10.2)	97.1 (89.8–99.6)	0.729
	3–5 (*n* = 170)	4.7 (2.1–9.1)	95.3 (90.9–97.9)	
Length of stay in minutes	Mean	380 (326–433)	305 (285–325)	**0.013**
ED discharge destination (%)	Discharge (*n* = 81)	4.3 (1.2–10.5)	95.7 (89.5–98.8)	0.687
	Admitted (*n* = 94)	2.5 (0.3–8.6)	97.5 (91.4–99.7)	
	Other^a^ (*n* = 63)	6.3 (1.8–15.5)	93.7 (84.5–98.2)	

*Note:* Differences between groups were tested using Welch's *t* test for continuous variables (age; length of stay), Fisher's exact test was used for categorical variables. For ED discharge destination, only discharged versus admitted was tested. Bold indicates significant *p* value (*p* < 0.05).

^a^ Transferred for further assessment.

### 3.2 Qualitative Analysis Results

Nine registered and clinical nurses participated in the study, with professional experience ranging from less than 1 year to 15 years (Table S4). Only one participant reported substantial aged care experience, specifically in nursing homes and dementia wards. Several others had limited or indirect exposure to aged care settings, such as student placements or work in units with a higher proportion of older patients. The majority had no direct aged care experience.

#### 3.2.1 Clinical Identification of Delirium Risk

Participants described a multifactorial approach to identifying delirium risk, drawing on clinical cues such as altered GCS scores, behavioural changes and collateral history. Establishing a cognitive baseline, often sourced from family or aged care facilities, was viewed as essential (Table 3, quote 1). Commonly recognised risk factors included age, infection (particularly sepsis) and cognitive impairment (Table 3, quote 2).

**TABLE 3:** Illustrative quotes organised by theme and participant.

Theme	Quote	Participants
Clinical identification of delirium risk	1: ‘Gathering sort of collateral… to see… is this out of the ordinary for them to be behaving this way’.	P1
	2: ‘Age is already a risk… we see a lot of septic or infectious patients who pose risk for delirium’.	P3
	3: ‘You normally pick up pretty quickly if someone's on the same wavelength or not’.	P1
	4: ‘New confusion… aggressive behaviour or agitation… makes me want to investigate further’.	P2
Use and integration of screening tools	5: ‘We do have the 4AT… I am not the best at remembering to do that’.	P7
	6: ‘Can I say instinct?… I just get a feeling sometimes’.	P5
	7: ‘I don't think we have much informed in terms of like a standard’.	P2
	8: ‘Like a sepsis pathway? That's actually the only one I really know that kind of talks about delirium’.	P3
Barriers to screening	9: ‘It's not extremely high on my priority… it can get pushed back’.	P6
	10: ‘It's loud, it's noisy… some patients might score differently because they are in an emergency department’.	P2
	11: ‘We shouldn't be leaving it to [the FRAIL team], but it does often get left to them’.	P7
	12: ‘I definitely don't think it's mandatory that we do any sort of delirium screening stuff’.	P1
	13: ‘I don't know which standards apply’.	P6
Interdisciplinary collaboration and communication	14: ‘They always manage to pick up things that the rest of us haven't. They're really good’.	P8
	15: ‘The actual sticker itself is extremely helpful… a visual tool to know that it's been done’.	P6

Less frequently cited factors included falls, medication effects (mainly opioids), and sensory deficits. Nurses often relied on instinct and informal observation, noting subtle behavioural or communication changes as potential indicators (Table 3, quote 3). Despite this awareness, identification was typically reactive rather than systematic, with screening often initiated in response to overt behavioural changes or observable decline rather than as part of routine assessment (Table 3, quote 4).

#### 3.2.2 Use and Integration of Screening Tools

The 4AT was the most frequently mentioned tool, valued for its speed, though its use was inconsistent due to forgetfulness, time pressure or competing priorities (Table 3, quote 5). Many nurses instead relied on clinical judgement and informal conversation or observation to identify confusion or disorientation (Table 3, quote 6).

The GCS was widely used as a measure of altered mental status, sometimes paired with the 4AT or used prior to additional assessment. Screening was often postponed until baseline cognition could be confirmed, with collateral information from family, aged care facilities and handover seen as key facilitators (Participants 1, 2, 3, 6). Participants also reported uncertainty around standardised protocols and insufficient prompts in documentation systems, identifying a need for clearer processes and better integration of tools into routine workflows (Table 3, quote 7). Structured pathways, such as sepsis protocols or specialist involvement (e.g., FRAIL team), were considered helpful but limited (Table 3, quote 8). While the FRAIL team included delirium screening in broader assessments (Participants 3, 5, 6), their involvement sometimes blurred primary nursing responsibilities.

#### 3.2.3 Barriers to Screening

Time pressure was the most frequently cited barrier, with the 4AT often deprioritised among competing demands (Table 3, quote 9). Environmental challenges, including noise, overstimulation and lack of privacy, were seen as reducing both feasibility and accuracy (Table 3, quote 10). Additional barriers included patient agitation, difficulty engaging in assessments and uncertainty about baseline cognition. Tool‐related issues such as difficulty recalling 4AT questions and lack of digital integration also hindered use. Role ambiguity further contributed, with screening sometimes deferred to specialist teams instead of being embedded in nursing practice (Table 3, quote 11).

System‐level supports were described as inconsistent. Education and training were identified as key gaps; while some participants recalled past in‐services or student experiences, most described formal learning as optional, outdated or absent from mandatory programmes, contributing to poor awareness of the Delirium Clinical Care Standard (Table 3, quotes 12, 13). Staffing issues, especially the need for one‐on‐one care, were also noted, and while AINs and float nurses could help, access was variable.

#### 3.2.4 Interdisciplinary Collaboration and Communication

Participants reported mixed experiences with interdisciplinary communication. Collaboration with specialist teams, particularly the FRAIL team, was consistently valued for their expertise and proactive patient support (Table 3, quote 14). However, reliance on these teams sometimes contributed to communication delays and uncertainty around primary nursing roles. Informal communication, especially verbal handover, was commonly used to flag concerns when documentation was limited.

Physical prompts such as 4AT stickers were seen as useful for signalling completed assessments (Table 3, quote 15). Conversely, participants reported limited awareness or use of digital tools for communication or documentation, noting an absence of integrated systems or real‐time alerts and highlighting broader gaps in digital support and training.

### 3.3 Integration of Quantitative and Qualitative Findings

**FIGURE 1:**
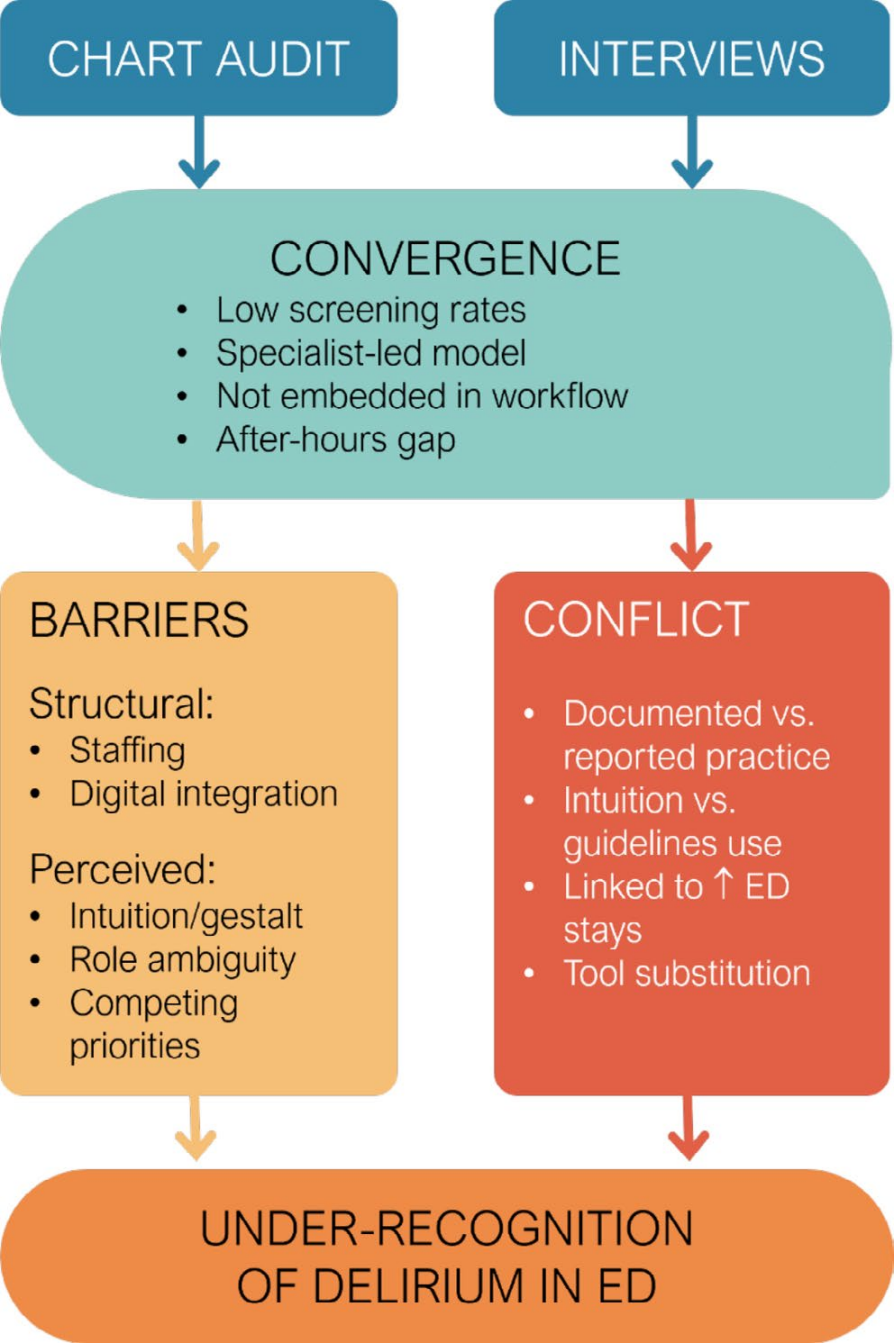
Conceptual process map of delirium screening in the emergency department (ED). This diagram integrates findings from chart audits and qualitative interviews. Convergence highlights shared observations of low screening rates, specialist‐led model of care, lack of integration into routine workflows and gaps in after‐hours screening. Conflict represented discrepancies between documented and reported practice, including reliance on intuition over guideline‐based tools, use of informal or substitute assessments and exploratory associations between screening and ED length of stay. Structural system constraints (e.g., staffing constraints, limited digital integration) and perceived cultural barriers (e.g., role ambiguity, reliance on intuition) contribute to the systemic under‐recognition of delirium. The map highlights the need to embed guideline‐aligned screening into routine ED practice.

Convergence between quantitative and qualitative data was found in the low screening rates and the heavy reliance on specialists. Qualitative accounts of staffing constraints and limited after‐hours specialist access explained the quantitative absence of night and weekend screening (Figure 1). Similarly, the concentration of screening within weekday hours aligned with reported reliance on the FRAIL team, whose availability was largely limited to business hours, reinforcing the dependence on specialist‐led assessment.

Conflict was noted regarding GCS use; nurses frequently used it as a proxy for cognitive assessment, despite it not being a validated delirium tool. While the audit indicated a lack of documented screening with the 4AT, interviews indicated general awareness of the tool and its value while relying on intuition and informal observation due to time pressures and environmental challenges. This divergence suggests that although knowledge of delirium risk factors and screening tools exists, translation into consistent practice remains limited. Reliance on informal assessment further elucidates the discrepancy between low documented screening rates and clinicians' perceived recognition of delirium risk, suggesting that elements of assessment may occur without formal documentation.

The observed difference in ED LOS between screened and unscreened patients highlights divergence: quantitatively, screened patients had significantly longer lengths of stay, although this finding should be interpreted with caution given the small number of screened patients. Qualitatively, nurses described screening as deprioritised amid competing demands, raising questions about whether screening is more likely to occur in patients already requiring extended care, where time permits more comprehensive assessment.

**FIGURE 2:**
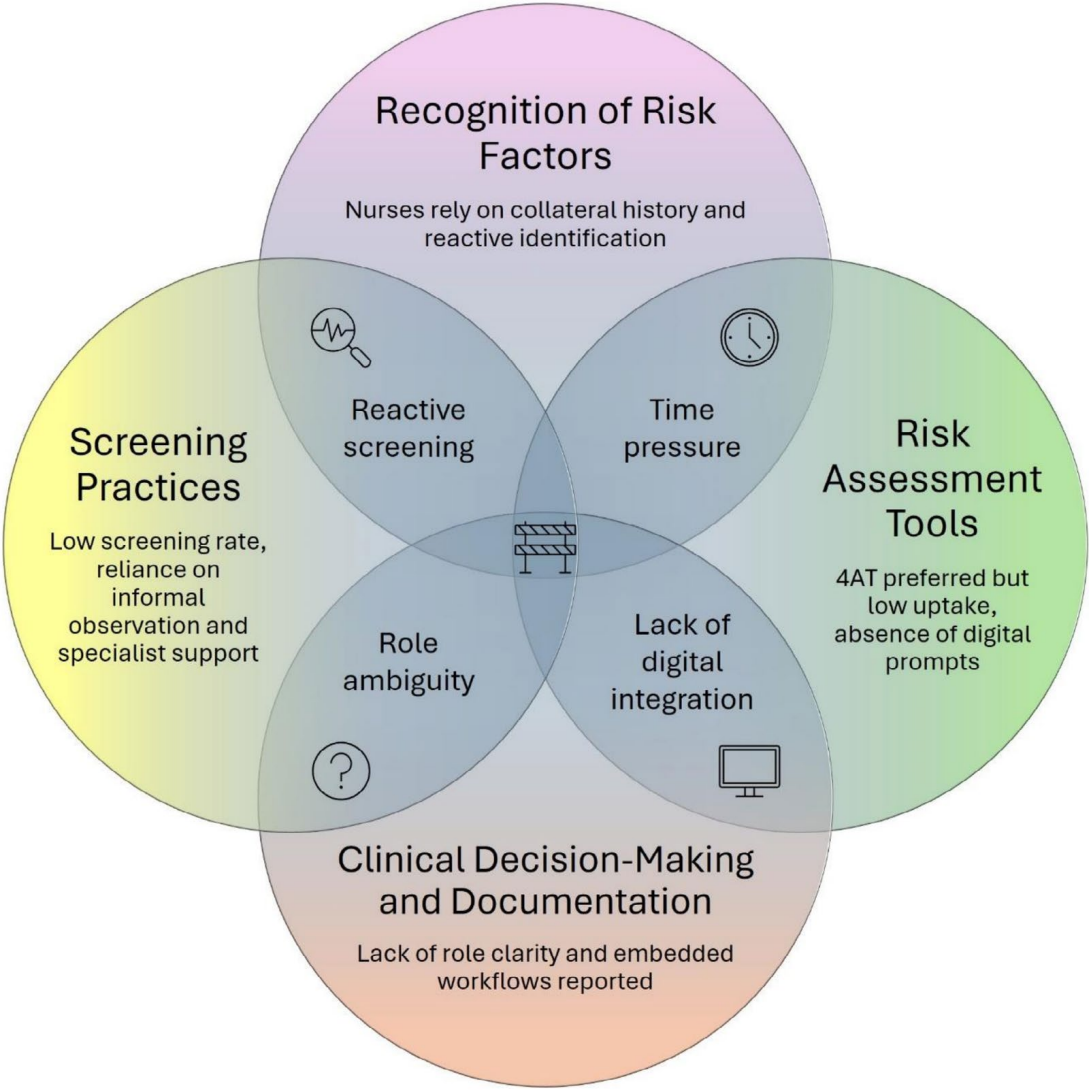
Parent themes and overlapping barriers to delirium screening in the emergency department. The Venn diagram illustrates four key domains—Recognition of Risk Factors, Risk Assessment Tools, Screening Practices and Clinical Decision‐Making and Documentation—and their intersections. Shared challenges include reactive screening, role ambiguity, time pressure and lack of digital integration, highlighting systemic and cultural barriers to guideline‐aligned practice.

Additional convergence was noted in patient characteristics: screened patients were older and more likely to arrive by ambulance, aligning with qualitative findings that clinicians prioritised screening in patients perceived to be at higher risk, such as those with more acute presentations, frailty or complex clinical needs. Taken together, integrated findings identified both structural barriers (e.g., staffing, digital integration, documentation systems) and cultural factors (e.g., reliance on intuition, role ambiguity) as barriers to delirium screening in the ED (Figure 2).

## 4 Discussion

This study examined emergency nurses' current practices in recognising delirium risk and conducting screening, mapped to Statements 1 (Recognising Risk) and 4 (Delirium Assessment) of the Australian Delirium Clinical Care Standard [12]. Findings revealed a substantial gap between national expectations and ED practice, with only 4.2% of eligible patients screened, consistent with the limited uptake reported in other ED studies [4, 6].

Although nurses readily identified common risk factors, such as age, cognitive impairment and infection, delirium screening remained reactive and specialist‐led, rather than embedded into routine nursing assessment. Similar to Schonnop et al. [19], the GCS was frequently used as a proxy for cognitive assessment, despite not being validated for delirium detection and not recommended in NICE or PADIS guidelines, which instead endorse tools such as the 4AT and CAM [20, 21]. This reliance on GCS and clinical intuition is likely to capture hyperactive presentations while hypoactive delirium remains under‐recognised.

The 4AT was widely regarded as the preferred screening tool, aligning with strong evidence supporting delirium risk factors and the diagnostic accuracy and efficiency of screening tools such as the 4AT [22, 23, 24]. However, its application was inconsistent and typically triggered by overt behavioural changes or clinical suspicion, echoing previous reports of emergency clinicians' reliance on informal assessments [19, 25]. This trust in informal assessment may also contribute to under‐documentation of delirium screening, meaning that structured screening rates derived from audit data may underestimate actual clinical assessment. Among patients screened in this study, most recorded a 4AT score of 0, with only two scoring 4, raising questions about whether clinical triggers reliably identify those most at risk. The evidence linking universal routine delirium screening in the ED to improved patient outcomes, nevertheless, remains less direct. This highlights the importance of considering implementation context and feasibility when interpreting screening practices in real‐world clinical settings.

Study participants frequently framed delirium risk within the context of sepsis pathways, an area where structured, well‐implemented guidelines have achieved strong adherence [26, 27]. Yet other risk factors such as sensory impairment and medication effects were under‐recognised, emphasising the need for ongoing national initiatives to support clinical effectiveness. Persistent barriers to screening, including time pressure, environmental disruption, competing priorities and uncertainty regarding baseline cognition, reflect largely perceived barriers that influence clinicians' ability to prioritise screening within busy ED workflows [28, 29, 30].

In contrast, structural barriers were evident in system‐level limitations, including uncertainty around standardised protocols and limited integration of prompts within documentation systems. While informal handovers and visual cues (such as 4AT stickers) were helpful, education and training were widely reported as outdated, optional or absent from mandatory programmes—a significant limitation given evidence that electronic integration and leadership support are key to successful screening implementation [31, 32]. The absence of integrated digital prompts or automated reminders represents a key structural barrier, limiting opportunities to embed screening into routine workflows.

System‐level supports such as the FRAIL team were viewed positively and were responsible for most screenings, consistent with studies demonstrating improved detection when specialist teams are involved [33]. However, this reliance generated role ambiguity, delays and inconsistent uptake, particularly outside weekday business hours. Screening was absent on nights and weekends, reflecting findings by Meged‐Book et al. that detection rates decline when dedicated evaluators are unavailable [34]. In this study, delirium screening was an established referral criterion for the FRAIL team; however, the concentration of screening within this service suggests a potential unintended shift from a referral‐based model to one in which screening responsibility was implicitly transferred to specialist staff. This may have reduced the integration of screening into routine nursing workflows and contributed to its limited uptake outside specialist availability. These findings highlight a structural dependency on specialist teams rather than distributed responsibility within routine care processes, which becomes increasingly unsustainable with an ageing population.

Distinguishing between perceived and structural barriers has important implications for implementation, as structural constraints may be more amenable to system‐level interventions such as workflow redesign and digital integration, whereas perceived barriers may require targeted education, role clarification and cultural change.

## 5 Conclusions

Overall, this study highlights that reliance on specialist availability may limit consistent implementation of delirium screening as part of core ED practice. The findings suggest that more decentralised approaches to screening, embedded within routine nursing workflows, may support improved adherence to national standards. Achieving this may require clearer role delineation, enhanced education and greater integration of digital prompts within electronic health records to support systematic assessment for older patients.

## 6 Limitations

This study has several limitations that should be considered when interpreting the results. First, this single‐site study at a metropolitan hospital may not reflect practice in rural or private settings. The study site is a large teaching and referral centre typical of major Australian EDs. Geriatric‐focused ED service models, such as the FRAIL team, are consistent with broader Geriatric Emergency Department Intervention (GEDI) approaches implemented across Australian healthcare settings [35]. However, variation in local workflow design and digital system integration remains common. In this study, the absence of fully integrated electronic prompts may have contributed to reliance on specialist teams and informal screening practices. These contextual factors should be considered when assessing the transferability of findings to other settings.

Second, we assessed only two statements of the *Clinical Care Standard*; broader aspects of prevention and management were not evaluated. Additionally, it is possible that delirium screening occurred but was not documented in the medical record. As the audit relied on recorded use of the 4AT, undocumented assessments or informal clinical evaluations would not have been captured, potentially leading to an underestimation of true screening rates, particularly given qualitative findings that clinicians often relied on informal assessment rather than structured tools. The small number of patients who were screened (*n* = 10) consequently limits the reliability of statistical comparisons, and any observed associations should be interpreted as exploratory rather than definitive. Similarly, the small qualitative sample, albeit having reached saturation, may have limited the depth and diversity of perspectives, and findings may not fully capture the range of experiences across the ED workforce. Furthermore, as interviews were offered on a first‐come basis, it is possible that more motivated or engaged staff self‐selected to participate, introducing potential selection bias and limiting the representativeness of perspectives captured. Finally, while the mixed‐methods design provided a comprehensive overview of current practices, the integration was descriptive rather than explanatory, limiting the interpretation of causative relationships between identified barriers and screening rates.

## Funding

This work was supported by the Emergency Medicine Foundation (EMCB‐332R39‐2023‐JORDAN) and the Prince Charles Hospital Foundation (PCHCollab2024‐12).

## Conflicts of Interest

The authors declare no conflicts of interest.

## Supporting information


**Table S1:** Interview guide questions and relation to mapped themes.
**Table S2:** Mapping of key concepts to relevant interview questions and resulting thematic codes for analysis in NVivo. For each code, the description, inclusion and exclusion criteria used to develop the NVivo Codebook are provided.
**Table S3:** Number of references identified for each code in each of the nine interviews (I).
**Table S4:** Interview participant characteristics.

## Data Availability

The data that support the findings of this study are available on request from the corresponding author. The data are not publicly available due to privacy or ethical restrictions.
