# Supplementary material for: Delirium Screening in the Emergency Department: Barriers, Enablers and Alignment With Clinical Standards—A Mixed‐Methods Study
Source: Emerg Med Australas. 2026 Jun 17;38(3):e70303. doi: 10.1111/1742-6723.70303 (PMC13273580; doi:10.1111/1742-6723.70303)
Supplement: Supplementary file 1 — Table S1: Interview guide questions and relation to mapped themes. Table S2: Mapping of key concepts to relevant interview questions and resulting thematic codes for analysis in NVivo. For each code, the description, inclusion and exclusion criteria used to develop the NVivo Codebook are provided. Table S3: Number of references identified for each code in each of the nine interviews (I). Table S4: Interview participant characteristics. [file EMM-38-0-s001.docx]

# Supplementary Materials

**Supplementary Table 1.** Interview guide questions and relation to mapped themes

| Interview Question | Mapped Theme |
| --- | --- |
| Can you share your experiences working with patients in the emergency department who may be at risk of delirium? | Recognition of Risk Factors |
| How do you typically approach identifying signs of delirium in your patients? | Delirium Screening Practices |
| What methods, tools, or informal practices do you or your colleagues use to screen for delirium? | Use of Screening Tools |
| Can you describe the current standard practice for delirium screening in our ED? Could you share an example where you used a screening tool? | Delirium Screening Practices |
| Is there anything that influences your decision to use, or not use, a screening tool? | Recognition of Risk Factors |
| Is there anything that makes delirium screening more difficult in the ED? | Barriers to Delirium Screening |
| Can you think of specific environments, organizational support or staffing that makes it more difficult to screen? | Barriers to Delirium Screening |
| Is there anything specific to the patient that influences if or how you screen for delirium? | Recognition of Risk Factors |
| What physical resources (e.g. equipment, tools, space) do you have available for delirium screening? | Organisational Support |
| What resources, for example staff, training, education helps you feel prepared to identify delirium in your patients? | Organisational Support |
| Are you aware of clinical standards, protocols, or pathways that guide or support you with screening for delirium? | Organisational Support |
| Do you have any thoughts on the usefulness of digital health tools to support delirium screening? | Communication and Collaboration |
| Are there any changes in your workplace or workflow that would make it easier to incorporate delirium screening into routine care? | Delirium Screening Practices / Organisational Support |
| Do you have ideas to improve delirium screening for patients in your ED? | Delirium Screening Practices |
| What collaboration with other healthcare professionals do you utilize for delirium screening? | Communication and Collaboration |
| How does collaboration with other healthcare professionals, for example geriatricians, occupational therapists and other impact your delirium screening? | Communication and Collaboration |

**Supplementary Table 2.** Mapping of key concepts to relevant interview questions and resulting thematic codes for analysis in NVivo. For each code, the description, inclusion and exclusion criteria used to develop the NVivo Codebook are provided.

| Parent Theme | Code (Node Name) | Description | Inclusion | Exclusion |
| --- | --- | --- | --- | --- |
| Recognition of Risk Factors | Awareness of Delirium Risk | Nurses understanding of delirium risk factors and the importance of early identification. | Mentions of risk factors, patient related factors, or personal knowledge. | General comments not related to delirium risk. |
| Risk Assessment Tools | Use of Screening Tools | Use and perception of validated tools for identifying patients at risk of delirium. | References to specific tools (e.g., 4AT, CAM), protocols, or assessments. | Informal assessments or general observations without tool use. |
| Screening Practices in ED | Delirium Screening Practices | How and when nurses screen for delirium in the emergency department. | Timing of screening, triggers (e.g., presentation, change in condition). | Screening outside the ED or unrelated cognitive assessments. |
| Screening Practices in ED | Barriers to Delirium Screening | Challenges nurses face in conducting delirium screening. | Time constraints, staffing issues, lack of training, or tool accessibility. | Barriers unrelated to delirium screening. |
| Clinical Decision-Making and Documentation | Communication and Collaboration | How delirium screening and risk are documented and communicated across teams. | Notes in patient records, handover protocols, interdisciplinary communication. | Informal discussions not tied to documentation. |

**Supplementary Table 3.** Number of references identified for each code in each of the nine interviews (I).

| Code | I1 | I2 | I3 | I4 | I5 | I6 | I7 | I8 | I9 | Total |
| --- | --- | --- | --- | --- | --- | --- | --- | --- | --- | --- |
| Recognition of Risk Factors | 5 | 5 | 5 | 4 | 4 | 4 | 2 | 2 | 2 | 33 |
| Risk Assessment Tools | 3 | 3 | 3 | 3 | 3 | 3 | 3 | 2 | 2 | 25 |
| Screening Practices in ED | 6 | 6 | 6 | 6 | 6 | 6 | 3 | 3 | 3 | 45 |
| Barriers to Delirium Screening | 5 | 6 | 4 | 4 | 4 | 5 | 4 | 3 | 3 | 38 |
| Communication & Collaboration | 4 | 4 | 3 | 3 | 4 | 4 | 3 | 3 | 3 | 31 |

**Supplementary Table 4.** Interview participant characteristics

| Participant | Sex | Role | Years of Experience | Aged Care Experience |
| --- | --- | --- | --- | --- |
| P1 | F | Registered Nurse | 15 | 2 years in nursing homes, casual pool including dementia wards |
| P2 | M | Registered Nurse | 8 | Experience with geriatric patients in Rapid Access Medical and Surgical and Early Assessment Units. |
| P3 | F | Registered Nurse | 1.5 | No |
| P4 | F | Registered Nurse | 4 | No |
| P5 | F | Registered Nurse | 3.5 | Only ED short stay |
| P6 | F | Clinical Nurse | 8 | Limited – part of the Older Persons Assessment and Liaison Service for a couple of months |
| P7 | F | Clinical Nurse | 8 | No |
| P8 | F | Registered Nurse | <1 | Only University placements |
| P9 | F | Registered Nurse | 7 | No |
